# Carriers Based on Zein-Dextran Sulfate Sodium Binary Complex for the Sustained Delivery of Quercetin

**DOI:** 10.3389/fchem.2020.00662

**Published:** 2020-09-30

**Authors:** Tian-xing Wang, Xiao-xi Li, Ling Chen, Lin Li, Srinivas Janaswamy

**Affiliations:** ^1^Ministry of Education Engineering Research Center of Starch and Protein Processing, Guangdong Province Key Laboratory for Green Processing of Natural Products and Product Safety, School of Food Science and Engineering, South China University of Technology, Guangzhou, China; ^2^Department of Dairy and Food Science, South Dakota State University, Brookings, SD, United States

**Keywords:** quercetin, zein, dextran sulfate sodium, colloid particles, sustained release, Higuchi model

## Abstract

Herein, a self-assembly formulation of Zein and dextran sulfate sodium (DSS) binary complex has been developed for the quercetin (Que) delivery. The prepared particles display a smooth sphere in the range of 180 ~ 250 nm. The addition of DSS shields the Trp residues of Zein that were located on the hydrophilic exterior and in-turn reduces the surface hydrophobicity of the nanoparticles. The presence of DSS, indeed, increases the encapsulation efficiency of Que from the initial 45.9 in the Zein to 72.6% in the Zein/DSS binary complex. A significant reduction of Que diffusion in the simulated intestinal conditions has been observed with the addition of DSS on the nanoparticles, which also improves Que bioavailability. The release mechanism of Que-loaded Zein/DSS composites is in accordance with the Higuchi model (Q = 0.0913t^0.5^+0.1652, *R*^2^ = 0.953). Overall, nanoparticles based on Zein-DSS complexes stand out as an attractive carrier system of quercetin and the outcome could be extended to several bioactive compounds.

**Graphical Abstract:**
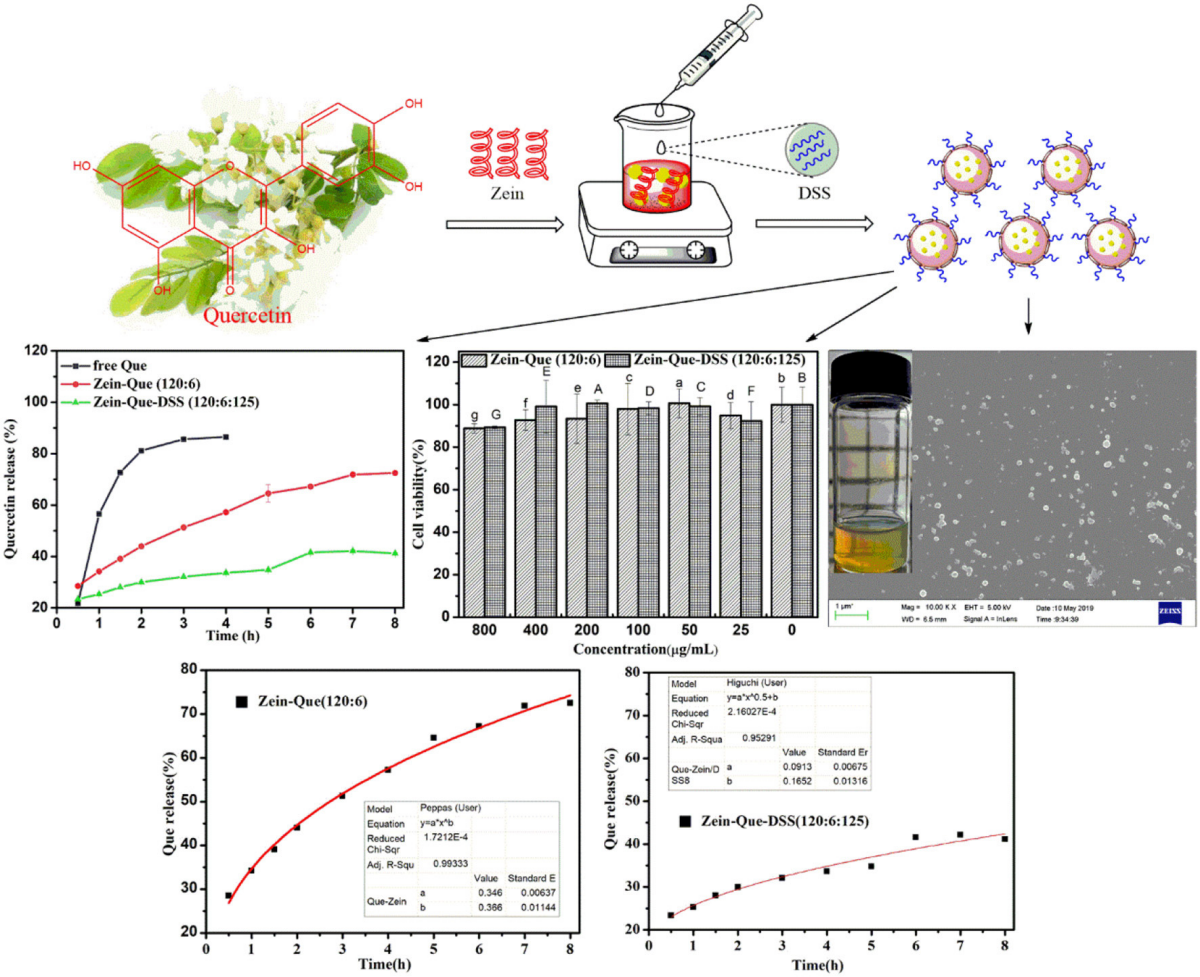
Quercetin-loaded Zein/DSS biopolymeric colloidal particles with high-performance capacity of encapsulation and forceful sustained-release in the manner of the Higuchi model (Q = 0.0913t^0.5^+0.1652, *R*^2^ = 0.953).

## Introduction

Quercetin (Que) is one of the important natural flavonoids and presents commonly in several medicinal plants, fruits, and vegetables (Hertog, 1992). It displays widespread pharmacological and physiological activities including anti-hypertensive (Andres et al., 2018), anti-viral (Danciu et al., 2016), anti-inflammatory (Yao et al., 2016), anti-oxidant (Bustos et al., 2016), and anti-tumor (Sun et al., 2013) properties. Its protective ability against acute myocardial infarction and heart disease (Patel et al., 2018) along with lowering transaminase function and alleviating allergy symptoms (Ding et al., 2019) is gaining further attention as a valuable medicine. More importantly, quercetin's propensity to retard glucagon-like peptide (GLP-1) release and to restore target cell sensitivity to insulin (Denise et al., 2011; Kunasegaran et al., 2016) highlights it as a promising hypoglycemic compound to address type II diabetes. These potent properties, however, are limited due to inferior water-solubility and poor bioavailability of quercetin (Cai et al., 2013). In this regard, delivery systems that not only enhance water solubility but also improve bioavailability are needed. Advances in the design, development, and manufacturing of protein-based materials (Chen et al., 2006), polysaccharide-based fibers (Janaswamy and Youngren, 2012; Polowsky and Janaswamy, 2015), colloidal systems (Velikov and Pelan, 2008), emulsions (Ting et al., 2014), and nanoparticles (Yao et al., 2015) have laid out a strong foundation for nutraceutical delivery. In this long list, self-assembled biodegradable nanoparticles have gained popularity due to their versatility and indeed they do stand out as a viable delivery option for quercetin.

Nanoparticles exhibit significant superiority due to their ability for enhanced encapsulation and solubility of hydrophobic compounds (Tulin and Derman, 2018), suitable size for cellular uptake, viability for targeted delivery (Fang et al., 2010), and stable internalization capacity for clathrin-mediated endocytosis (Liu et al., 2018). Over the years, nanoparticles based on proteins and polysaccharides such as soy protein isolate (Yan et al., 2018), quinoa protein (Zheng and Gu, 2011; Qin et al., 2018), chitosan (Facchi et al., 2016), and Gum Arabic (Butstraen and Salauen, 2014) have gained widespread attention due to their intrinsic safety, biodegradability, and biocompatibility.

Zein is one of the plant-derived natural and inexpensive hydrophobic biomacromolecules. As a major storage protein in cereals, it gained a promising prospect as one of the suitable delivery systems (Lohcharoenkal et al., 2014). Its potent hydrophobicity, unique solubility, and resistance to gastric juices during digestion (Jiao et al., 2018) aid to develop micro- and nano-particles through self-assembly (Lei et al., 2018) as well as carriers with improved stability, increased solubility, prolonged drug circulation *in vivo*, reduced toxicity, and enhanced target distribution (Zhang et al., 2019). However, these particles are prone to aggregate and even precipitate at neutral to weak acidic pH conditions due to their protein origin and high hydrophobicity of wall-materials. Interestingly, sodium caseinate could stabilize Zein nanoparticles at low pH conditions (Patel et al., 2010) but such a state results in instable colloidal particles due to the vicinity of the sodium caseinate isoelectric point.

A strategic improvement of Zein nanoparticles would be to coat them with a layer of hydrophilic anionic polysaccharides (Hu et al., 2015). Toward this end, dextran sulfate sodium (DSS) is helpful, which is a polyanionic derivative of dextran produced through esterification with chlorosulfonic acid. Incidentally, DSS possess anti-inflammatory and antiviral activities (Santos et al., 2005; Romer et al., 2009). Its molecular weight ranges from 1.5 × 10^3^ to 5.0 × 10^5^ Da and is routinely used as an anticoagulant for clinical applications and immunity-associated studies. It is being explored as a model polysaccharide to induce inflammatory bowel disease (IBD) in biomedical/clinical applications (Takahara et al., 2010; Yan et al., 2019). It is also being employed as a stabilizer to protect natural sensitive bio-ingredients such as lapatinib (Mobasseri et al., 2017) and curcumin (Yuan et al., 2019). It has been successfully employed as a model drug carrier toward enhancing the stability of pre-existing drug delivery systems as well as gene delivery (Ruponen et al., 1999; Valente et al., 2013; Benhalima and Ferfera-Harrar, 2019). The selective binding capacity of DSS to cell-surface receptors further expands its potential for specific cell-targeting (Walton, 1952) including the binding of the quercetin uptake transporter namely multidrug resistance-associated protein 2 (MRP2) (Walgren et al., 2000). In this regard, we hypothesize that DDS could be an effective carrier material for quercetin.

The main objective of this research is to fabricate Que-loaded Zein/DDS colloidal nanoparticles using an antisolvent precipitation protocol. The physicochemical properties of the complexes along with intermolecular interactions between quercetin, Zein, and DSS have been investigated. In addition, particle morphology, quercetin encapsulation efficiency, and loading capacity have been established through FE-SEM and HPLC. The control-release behavior of quercetin in the phosphate buffer (pH 6.4) and toxicity for Caco-2 cell lines have been carried out using a dialysis bag and the Cell Counting Kit-8, respectively. Overall, DSS coating improves the structural stability and hydrophilicity of Zein nanoparticles. The outcome offers a value-added delivery system for quercetin and could well be extended to other bioactive and functional molecules toward improving stability and bioavailability.

## Materials and Methods

### Reagents and Chemicals

Dextran sulfate sodium (*M*_w_ = 6,500–10,000) was purchased from the Shanghai Aladdin Bio-Chem Technology Co., Ltd. (Shanghai, China). The Zein (~20 kDa) and quercetin (*M*_w_ = 302.24, purity ≥ 97.0%) were from the Shanghai Macklin Biochemical Co., Ltd. (Shanghai, China). The analytical-grade methanol, ethanol, hydrochloric acid, sodium hydroxide, HPLC-grade acetonitrile, and phosphate acid were from the Shanghai Aladdin Bio-Chem Technology Co., Ltd. (Shanghai, China). Citric acid, disodium hydrogen phosphate, sodium dihydrogen phosphate, and glacial acetic acid were of analytical grade.

### Fabrication of Quercetin-Loaded Zein/DSS Biopolymeric Nanoparticles

An antisolvent coprecipitation method was employed to fabricate Que-loaded Zein/DSS biopolymeric nanoparticles. Initially, precisely weighted amounts of Zein were dissolved in an aqueous ethanol solution (75% v/v) with an accurate concentration of 20 mg/mL and agitated magnetically with a stirring speed of 500 rpm for 1 h at room temperature. Thereafter, water-insoluble quercetin (0.16 g) was added and stirred for 1 h at 500 r/min. The formed solution was centrifuged to eliminate insoluble impurities. Subsequently, 40 mL of the resulting solution was dispersed dropwise in deionized water (160 mL) at pH 4.0 for 5 min under magnetic stirring (1,000 rpm) to obtain Que-loaded Zein nanoparticles. The ethanol was removed through vacuum-rotary evaporation (55°C, −0.09 mPa) and the removed solvent was replaced by equal parts of distilled water (pH = 4.0) to obtain 200 mL of Que-loaded biopolymeric dispersions and stored at 4°C. Parallelly, six conical flasks consisting of different concentrations of DSS solution (0.1, 0.5, 1, 1.5, 2, and 2.5 mg/mL) were prepared and stirred for 50 min. The pH was adjusted to 4.0 using a 1M solution of hydrochloric acid. Subsequently, part of the nanoparticle dispersions were diffused into the DSS solutions at a volume ratio of 4:5 (quercetin/Zein dispersions-DSS solution) and agitated during the self-assembly process with a magnetic stirrer (1,000 rpm) for 5 min. The dispersion was then centrifuged at 3,000 rpm for 10 min and colloidal dispersions were collected.

### Particle Size and Zeta Potential Determination

A Nano-ZetasiZer-MPT-2 instrument (Malvern Instrument, Ltd., Malvern, U.K.) was employed to measure the particle size, zeta potential, and polydispersity index (PDI) and Dynamic Light Scattering (DLS). All samples were diluted with acidic distilled water (pH = 4.0) at a ratio of 1:5 applicable for DLS measurements and were carried out in triplicate at 25°C.

### Fluorescence Spectroscopy and Differential Scanning Calorimetry (DSC) Analysis

The fluorescence of protein-polysaccharide complex nanoparticles was analyzed to estimate the binding stability of Que to nanoparticles using the fluorescence spectrophotometer (F-7000, Hitachi, Japan). The emission wavelength was in the range of 290–460 nm with an excitation wavelength of 280 nm and both the excitation and emission slit were set to 10 nm at a constant scanning speed (100 nm/min). Differential scanning calorimetry (DSC) studies were performed on 10 mg of the sample in flat-bottomed aluminum pans using the Perkin Elmer Pyris I DSC. The samples were heated from 30 to 230°C at a heating rate of 10°C/min. Inert atmosphere was maintained by nitrogen purging at a flow rate of 20 mL/min. The thermal curves were collected by the DSC-60 workstation.

### Field Emission Scanning Electron Microscopy (FE-SEM) Imaging and Circular Dichroism (CD) Analysis

The micro-configuration of freeze-dried sample powders was performed by a field emission scanning electron microscopy (FE-SEM, LEO 1530 VP, Germany) at an accelerating voltage of 5.0 kV and a working distance of 10 mm. Prior to that, a few powder samples were directly scattered upon conductively double-sided tape attached to a sample pan. The unattached powder was blown away with nitrogen and the primed samples were coated with a thin layer of gold to reduce or avoid the charged effect. Then the particle size of randomly selected Que-loaded Zein/DSS nanoparticles in the obtained FE-SEM images were calculated using the *Image J* software. An accounting report in the form of a histogram was converted to a curve diagram involving particle size distribution. Therein a peak value of the resulted curve was treated as the average particle size of the obtained nanoparticles. The CD spectra of all samples were performed under the following conditions (temperature 25 ± 1°C): far-UV range of 190–260 nm, bandwidth of 1.0 nm, recording speed of 50 nm, and optical path length of 0.1 cm. DICHROWEB, an online website with the URL of http://dichroweb.cryst.bbk.ac.uk, was used for the secondary structures (α-helix, β-sheet, β-turn, and random coil).

### X-Ray Diffraction (XRD) and Fourier Transform Infrared (FTIR) Spectroscopy Measurements

The x-ray diffraction patterns were recorded at 25°C using the Rigaku Full-Automatic Diffractometer (Rigaku, D/max-IIIA, Japan) with a copper target operated at 40 kV and 40 mA at wavelength 1.5418 Å. The intensity data were collected in the angular range of 3–60° of 2θ in a continuous scanning mode at a scan rate of 5°min^−1^. The chemical structures and intermolecular interaction of the composite nanoparticles and individual ingredients namely Zein, quercetin, and DSS were characterized by the Fourier transform infrared (FTIR) spectrophotometer (Nicolet, iS50 FT-IR, Thermo scientific, USA) equipped with an iD7 ATR fitting. The spectrum was collected in the wavenumber range of 500–4,000 cm^−1^ in a 64 scans mode at a resolution of 4 cm^−1^.

### Quercetin Encapsulation Efficiency (EE) and Loading Capacity (LC) Determination

The encapsulation efficiency (EE) and loading capacity (LC) of composite nanoparticles were estimated using the high-performance liquid chromatography (HPLC) (Rashidinejad et al., 2019). Initially, accurately weighted sample powders were re-dispersed in methanol and then followed 30 min of ultrasonic processing for complete dissolution. The content of Que in the methanol solution with a series of concentrations (0.5, 1, 2, 3, 4, 5, 6, 7, 8, 9, 10 mg/L) were detected to construct a standard curve (Y = 0.0604X+0.002, *R*^2^ = 0.9997). A Thermo U300 summit HPLC system equipped with a Poroshell 120 EC-C18 column (4.6 × 100 mm, 2.7 Micron) was used to analysis the amount of Que in nanoparticles using an isotropic mobile phase consisting of 50% (v/v) methanol and 50% (v/v) aqueous solution of phosphoric acid (0.2%, v/v). The flow rate, injection volume, and detection wavelength were 1 mL/min, 20 μL, and 358 nm, respectively. Finally, EE and LC were estimated using the following equations.

EE = content of Que in nanoparticles/total content of Que × 100%

LC = content of Que in nanoparticles/total weight of dry composites × 100%

### *In vitro* Quercetin Release Assay

With some modifications to the previously reported method (Liu et al., 2019), the release behaviors of free Que and Que extracted from Que-loaded Zein nanoparticles and Que-loaded Zein/DSS nano-complexes were evaluated in simulated intestinal fluid (pH = 6.4) conditions. Around 5 mg of the lyophilized sample was placed in a dialysis bag (*M*_w_ cutoff = 8,000 Da) and then submerged in 50 mL of artificial digestive juice (pH = 6.4) for 24 h with a constant incubation at 37 ± 0.5°C assisted by subtle magnetic stirring. Isometric incubation solutions (1.0 mL) were used to quantify the amount of released Que using HPLC at prescribed time intervals of 1 h and substituted by fresh digestive juice.

### Biocompatibility Evaluation

Using human colon adenocarcinoma cell line Caco-2 as testing material, a Cell Counting Kit-8 (CCK-8) was employed to evaluate cytotoxicity of the prepared nanoparticles. Initially, 100 μL of Caco-2 cell suspension was inoculated in a 96-well flat-bottom plate at a concentration of 3.5 × 10^4^ cells/well and incubated for 24 h at 37 ± 0.5°C in 5% CO_2_. Later, after removing the medium, cells were washed with 100 μL of sterile cold PBS once and sub-cultured in 100 μL of serum-free medium containing various concentrations (0, 25, 50, 100, 200, 400, 800 μg/mL) of nanoparticles for 24 h. The plates were slightly vibrated and incubated for 4 h after the addition of the CCK-8 solution (10 μL). The OD values were measured with a microplate reader at 490 nm.

### Statistical Analysis

All sample tests were implemented in triplicate, and results were represented as mean ± standard deviation (SD). Statistical differences were calculated by ANOVA using the SPSS 19.0 with Bonferroni correction, and *P* < 0.05 was deemed as statistically different.

## Results and Discussion

**Figure 1:**
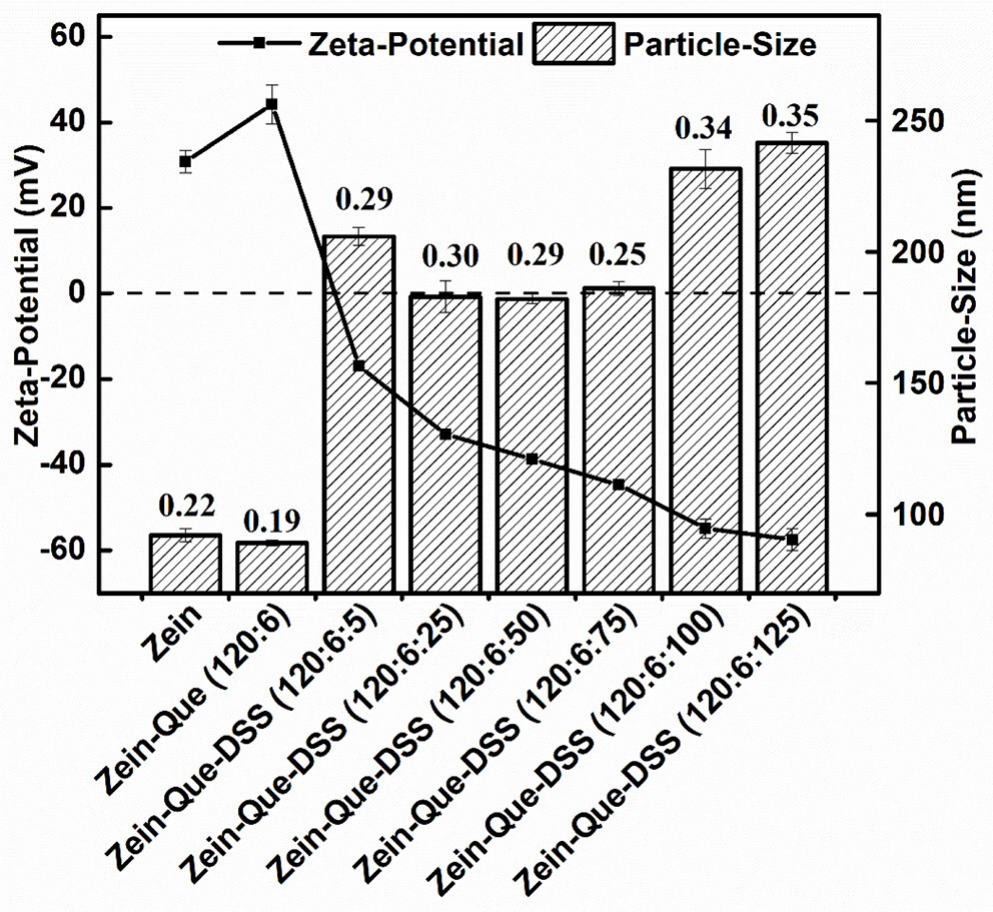
Particle size and zeta potential of Zein, Zein-Que, and Zein-Que-DSS complex nanoparticles, and therein the numbers represent the polydispersity index (PDI) of obtained colloidal particles.

The particle size, polydispersity index, and zeta potential of the prepared complexes are shown in Figure 1. The dimension of Que-loaded Zein nanoparticles was found to be around 89.1 nm (PDI = 0.19), which was smaller than that of the native Zein particles with a low PDI of 0.22. In contrast, the zeta potential of Zein nanoparticles increased from 30.8 to 44.3 mV, suggesting the formation of a diminutive and compact carrier structure (Patel et al., 2010). However, the addition of DSS (Zein-Que-DSS, 120:6:5) led to an increase in the particle size to 205.8 nm and an inverse transition of the surface charge peculiarity (−16.9 mV). Theoretically, the surface charge distribution of coated colloidal particles could be attributed to the charge of the outermost polymer (Irene et al., 2010). The drop in the potentials absolute value may be responsible for the increased particle size, which could be due to the reduction of electrostatic repulsion between ternary complexes leading to colloidal aggregates.

The particle size of the obtained composites decreased to 182.0 nm with a DSS concentration of 120:6:50, whilst potential increased from −16.9 to −38.7 mV. The increase in the surficial negative charge, indeed, resulted in the enhancement of electrostatic repulsion and thus suppressed the aggregation of nanoparticles. The DSS increase from 120:6:75 to 120:6:125 enlarged the particles from 186.0 to 241.4 nm (Figure 1) with a concomitant increase in the negative charge to around −57.5 mV. This could be attributed to the occurrence of cross-linking between sulfate groups and hydroxyl groups of DSS. This observation is in congruence with Zein-shellac particles that display shellac concentration dependent particle size (Chen et al., 2018). It appears that amino groups of proteins have electrostatic attraction preference to sulfated polysaccharides than that the carboxyl polysaccharides (Dickinson and Pawlowsky, 1996; Galazka et al., 1999) suggesting that DSS could be a favorable choice to enhance Zein nanoparticles. In summary, DSS deposition (120:6:125) resulted in the formation of high-density negatively charged Que-Zein/DSS nanoparticles with enhanced structural stability. Such nanoparticles have a diameter range of 200–500 nm and are suited well for the epithelial cells uptake (Walgren et al., 2000; des Rieux et al., 2005).

**Figure 2:**
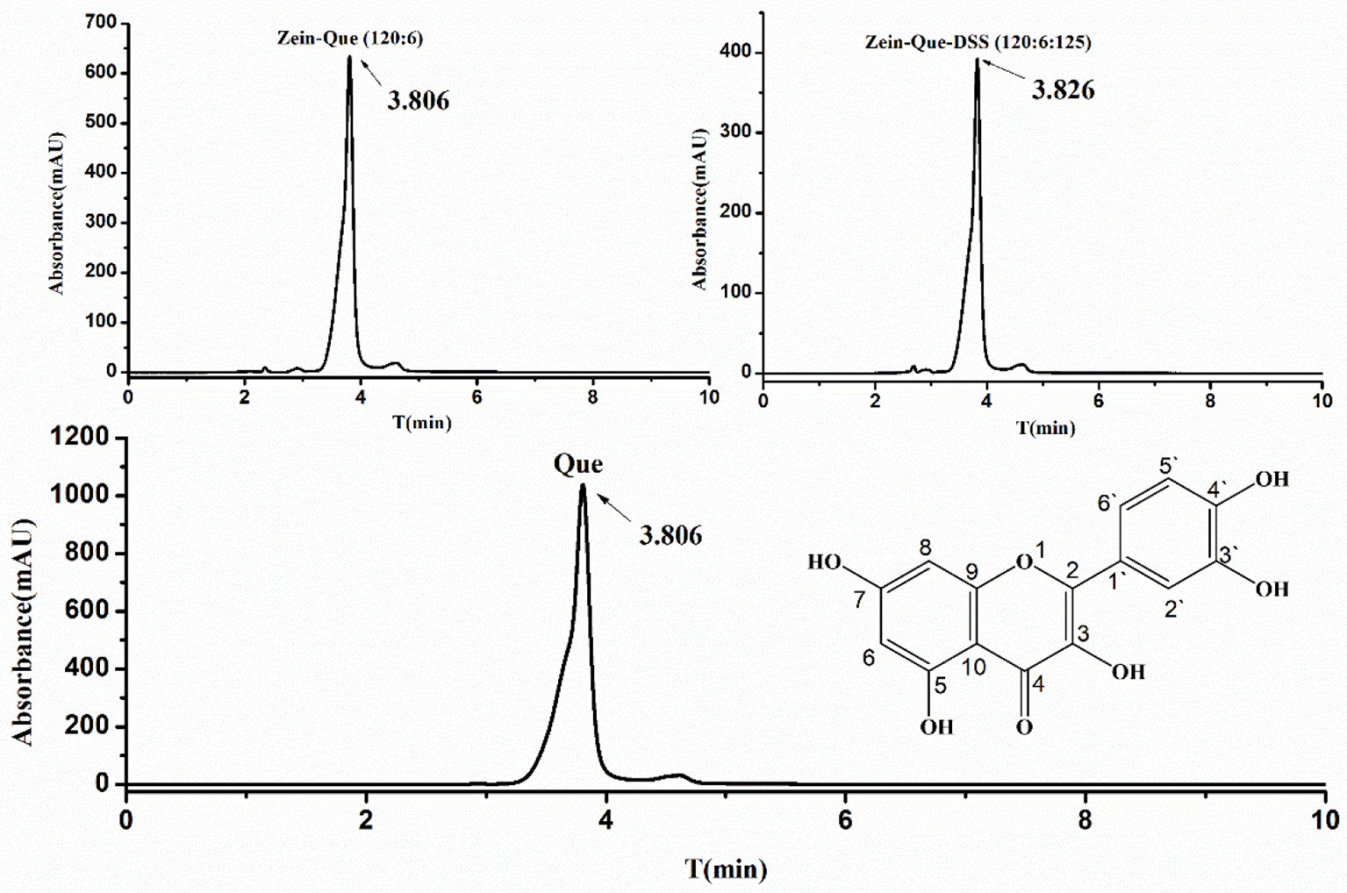
The chemical structural formula of quercetin, HPLC chromatogram of free Que, and Que supernatant obtained from Zein-Que (120:6) nanoparticles and Zein-Que-DSS (120:6:125) complex nanoparticles.

The characteristic chromatogram of free quercetin and quercetin extracted from Zein-Que (120:6) and Zein-Que-DSS (120:6:125) complex nanoparticles are shown in Figure 2. Based on the elution behavior and ultraviolet absorption performance, they all exhibited a single peak with a similar retention time of 3.806, 3.806, and 3.826 min, respectively. It appears that the vast majority of quercetin was entangled in the Que-loaded nanoparticles. The effects of the DSS addition on the EE and LC of nanoparticles are shown in Table 1. The presence of DSS led to a progressive increase of EE from 45.89% in Que-loaded Zein nanoparticles to 72.59% in the Que-loaded Zein/DSS ternary composites (120:6:125). One interesting finding was that the LC of Zein-Que-DSS nanoparticles gradually increased from 1.37 to 3.15%, in a concentration dependent way. Overall, favorable interactions between the hydrophobic protein Zein and sulfated polysaccharide DSS appear to facilitate the formation of a tightly structured nanoparticle that readily reflect with enhanced encapsulation efficiency and loading capacity of quercetin and could as well be applicable to other hydrophobic bioactive molecules.

**Table 1:** Encapsulation efficiency (EE) and loading capacity (LC) of quercetin supernatant obtained from Zein nanoparticles and Zein/DSS complex nanoparticles^1^.

**Samples**	**EE(%)**	**LC(%)**
Zein-Que = 120:6	45.89 ± 0.22^e^	2.16± 0.02^g^
Zein-Que-DSS = 120:6:5	64.50 ± 1.59^d^	1.37± 0.01^f^
Zein-Que-DSS = 120:6:25	65.19 ± 0.65^d^	2.52± 0.05^e^
Zein-Que-DSS = 120:6:50	67.09 ± 0.33^c^	3.03± 0.03^b^
Zein-Que-DSS = 120:6:75	67.79 ± 0.92^c^	3.15± 0.02^a^
Zein-Que-DSS = 120:6:100	70.02 ± 0.70^b^	2.87± 0.04^d^
Zein-Que-DSS = 120:6:125	72.59 ± 1.73^a^	2.95± 0.02^c^

1 *Results are means ± SD (n = 3). Different superscript letters in the same column represent significant differences (p < 0.05)*.

**Figure 3:**
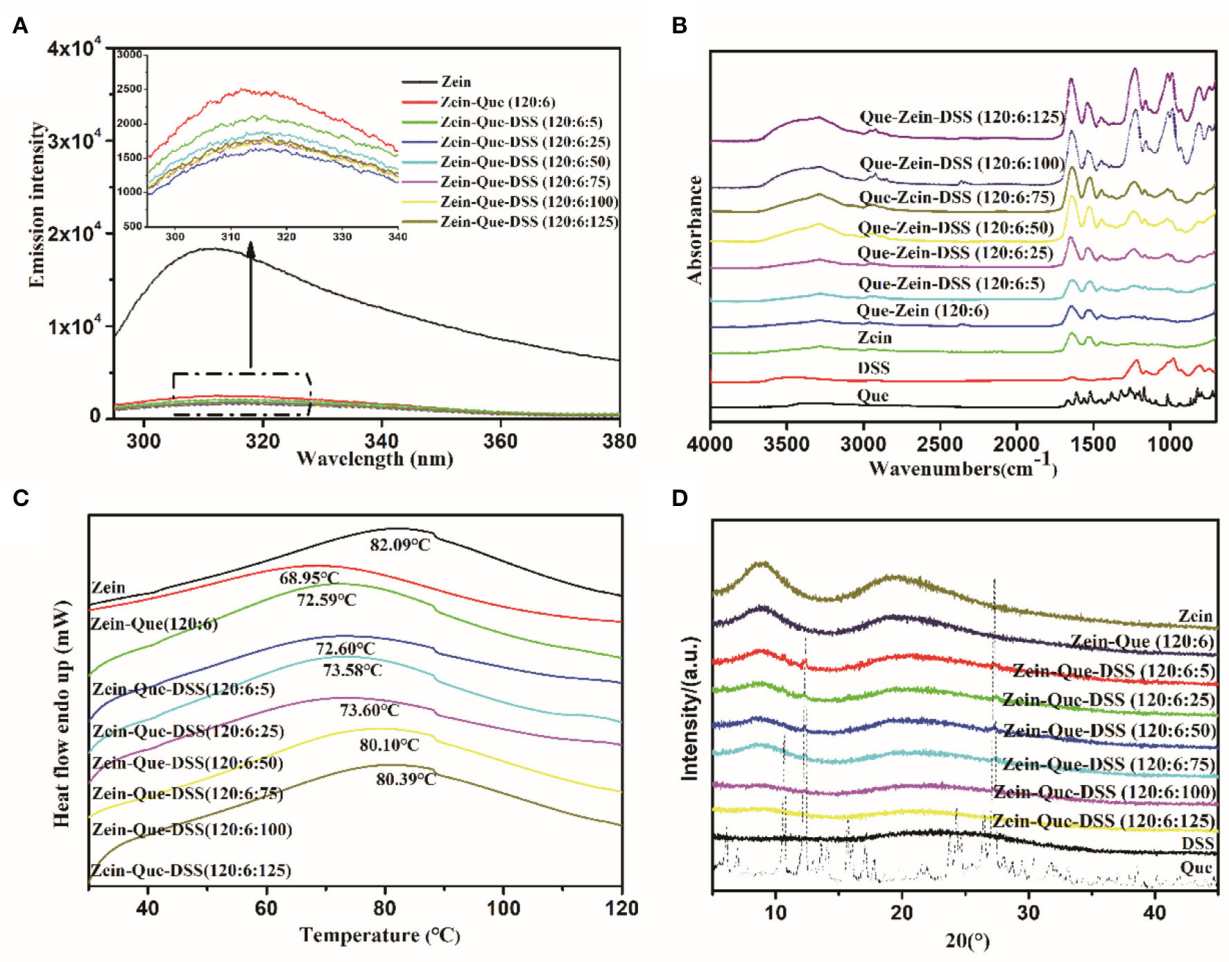
Fluorescence spectra **(A)**, FTIR **(B)**, DSC **(C)**, and XRD **(D)** of Que, Zein, DSS, Zein-Que nanoparticles, and Zein-Que-DSS complex nanoparticles.

The FTIR spectra of Que-loaded Zein nanoparticles bound with different concentrations of DSS are shown in Figure 3B. Quercetin displays characteristic peaks at 1664.3 cm^−1^ (C_4_ = O stretching), 1610.3 cm^−1^ (C_2_ = C_3_ stretching), 1382.7 cm^−1^ (C_3_-O-H phenolic bending) along with a peak at 3405.7 cm^−1^, which reveals the formation of intramolecular hydrogen bonding between -C_5_-O-H and -C_4_= O. The representative bands of DSS are present at 1218.8 cm^−1^ (SO_3_ antisymmetric stretching), 1006.7 cm^−1^, (C-O stretching), and 977.7 cm^−1^ (C-O-S antisymmetric stretching). The highlighted bands of Zein, parallel to -OH stretching, -C = O stretching (amide I), and -C-N stretching coupled with -N-H in-plane bending (amide II), are seen at 3293.9, 1637.3, and 1527.4 cm^−1^, respectively. Interestingly, the peaks that correspond to hydrogen bonds, amide I and amide II of Que-loaded Zein nanoparticles shifted to 3278.5, 1648.9, and 1535.1 cm^−1^ along with an increase in peak intensity. These are due to interactions between the amide groups of glutamine from Zein and hydroxyl groups from quercetin's aromatic ring. The intensity increase of individual bands could be due to the presence of a relatively high proportion of hydrophobic amino acids (leucine, alanine, and proline) in Zein. Further research, involving more Zein-like proteins is needed to establish this concept.

Interestingly, increasing DSS concentration shifted OH stretching of Que-loaded Zein nanoparticles from 3278.5 to 3282.3, 3291.9, 3288.0, 3288.0, 3290.0, and 3295.8 cm^−1^. Furthermore, movement of -SO_3_ and -C-O-S peaks from 1236.2 and 993.2 cm^−1^ to 1228.5 and 987.4 cm^−1^, respectively, due to electrostatic interactions, was also evidenced. Interestingly, for increased DSS amounts, e.g., 120:6:100 and 120:6:125, the peak intensity of the bands corresponding to -OH, amide I, amide II, -SO3, and -C-O-S stretching were significantly strengthened along with the increased negative charge density. These changes were due to possible hydrogen bonding and electrostatic interactions between Que and Zein/DSS. Similar non-covalent interactions were noticed in curcumin-loaded Zein nanoparticles coated with anionic polysaccharides pectin, CMC, and gum Arabic (Chao et al., 2017).

The conformational changes of Que-loaded Zein nanoparticles subject to the deposition of DSS have been analyzed through fluorescence spectra and circular dichroism. As the dominant fluorophore of protein intrinsic fluorescence, Trp residues often serve as fluorescence probes to study the conformation transition of proteins entrapped in composite particles due to their hypersensitivity to microenvironment changes (Witthayaprapakorn, 2011). The effect of the DSS addition on the Trp fluorescence emission spectra of Zein nanoparticles is depicted in Figure 3A. It should be noted that Zein showed a maximum fluorescence emission at 312 nm after being excited at 280 nm. Once combined with quercetin, a dramatic fluorescence quenching occurred, presumably due to a number of intermolecular interactions consisting of collisional quenching, cross-relaxation, energy transfer, rearrangement, and ground-state complex formation (Joye et al., 2015). The fluorescence intensity of ternary composites gradually declined with an increase in DSS concentration from Zein-Que = 120:6 to Zein-Que-DSS = 120:6:25. Thus, it appears that the formation of particle aggregates along with dynamic collisional quenching results in shielding for the Trp residues (Yang et al., 2014). Interestingly, the fluorescence intensity of Zein-Que-DSS = 120:6:50 particles increased subtly compared to that of Zein-Que-DSS = 120:6:25 particles. The electrostatic repulsion and steric hindrance appear to inhibit the nanoparticles aggregation and in-turn expose Trp residues that were originally buried inside the hydrophilic core of Zein leading to an increase in the fluorescence intensity of nanocomplexes (Borges et al., 2012). These findings are in agreement with the particle size distribution. Increasing the DSS amount, the fluorescence intensity of ternary nanoparticles (Zein-Que-DSS = 120:6:75, 120:6:100 and 120:6:125) were gently inferior to that of Zein-Que-DSS nanoparticles (120:6:50).

Figure 3C presents the DSC thermogram of Zein powder and Que-loaded Zein complex nanoparticles coated with different concentrations of DSS. All the samples display a broader endothermic peak, which means a longer melting process, suggesting a non-crystalline state of the nanoparticles. The endothermic peak at 82.09°C was due to protein degradation and no phase transition was observed. The endothermic melting of Que-loaded Zein particles with the presence of DSS (120:6:5, 120:6:25) appeared at 72.59 and 72.60°C, respectively. It appears that particle size increase along with reduced surface charge density with DSS increase results in less stable particles that are non-resistant to heat. Interestingly, as the DSS amount increased the endothermic peak moved toward the alcohol soluble protein melting peak. Figure 3D illustrates the x-ray diffraction patterns of Zein, quercetin, DSS, and Que-loaded Zein/DSS nanoparticles. Sharp crystalline peaks at of 10.7, 12.4, 16.2, 23.8, and 27.3° were noticed for quercetin, however, completely a amorphous pattern was noticed for the nanoparticles. This suggests that the DSS coating might result in uniform distribution of quercetin in the nanoparticles. The loss of crystallinity was also noticed in the case of curcumin encapsulated Zein nanoparticles (Shaikh and Ankola, 2009; Patel et al., 2010).

**Figure 4:**
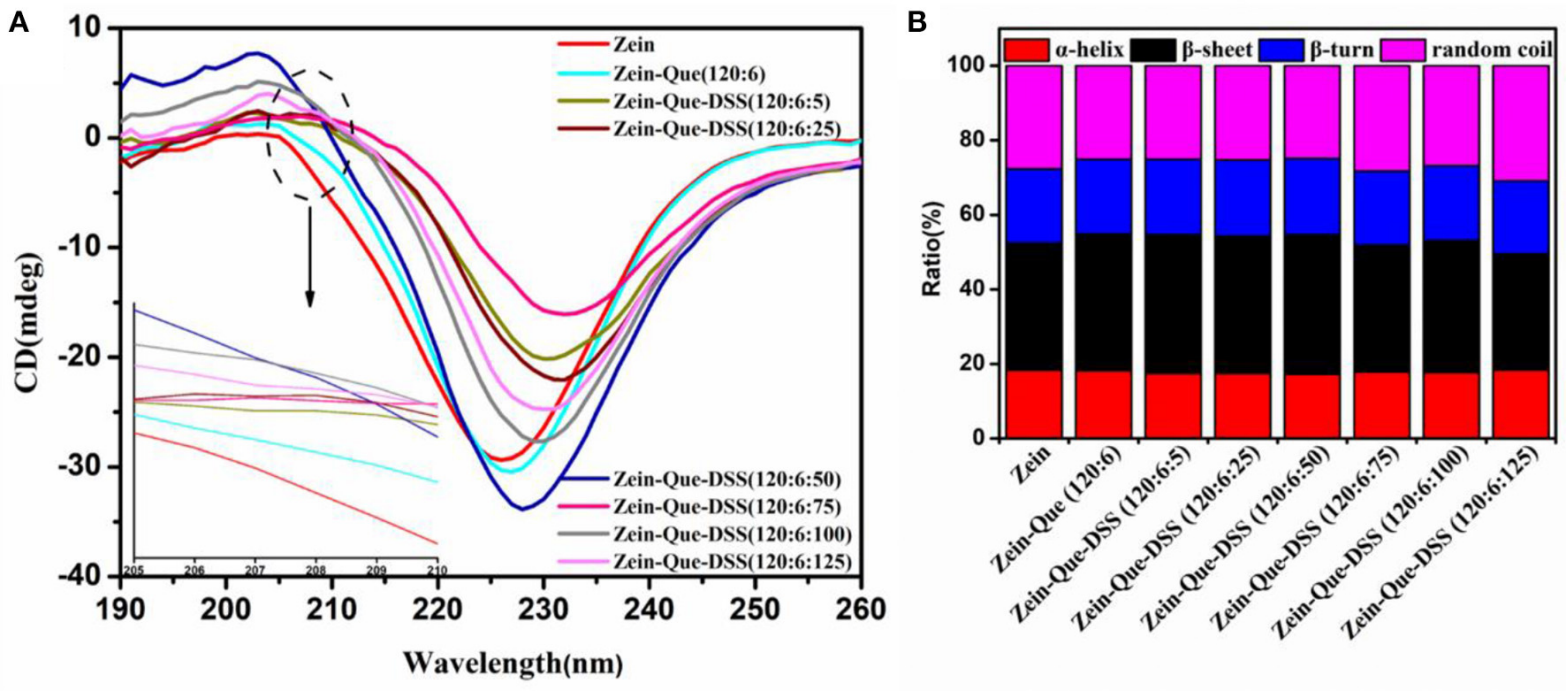
CD spectra **(A)** and proportion of secondary structures **(B)** of natural Zein and Zein situated in quercetin-loaded biopolymeric colloidal particles.

Changes in the secondary structure of Que-loaded Zein nanoparticles before and after the deposition of DSS are shown in Figure 4A. After complexing with quercetin and DSS, changes in the secondary structure of Zein were reflected with significant variations in the absolute values of mean residue ellipticities at 208 and 226 nm. In the spectra, presence of an α-helix yielded two negative peaks at 208 and 222 nm along with one positive peak at 192 nm and zero-crossing at 203 nm. Similarly, a negative band at 216 nm and a strong positive peak at 195 nm are characteristics of β-sheets. A negative band at around 180–190 nm and a positive band at 205 nm are due to β-turn but a negative peak at 198 nm and a positive peak at 200 nm are characteristics of random. Thus, it appears in the preset case, most of the changes were taking place in the α-helix. The negative band at 226 nm shifted to the direction of a longer wavelength with different CD intensities (Figure 4A), with the DSS increase, suggesting the occurrence of secondary structural changes in Zein.

The difference in proportions of the secondary structure of the obtained nanoparticles was calculated by the DICHROWEB procedure (SELCON3), and the results are shown in Figure 4B. The secondary structure of native Zein consisted of 18.3% α-helix, 34.1% β-sheet, 20.0% β-turn, and 27.6% random coil, which agrees with the literature (Sun et al., 2017) but with slight changes in the individual values. The differences could be due to variations in preparation methods of nanoparticles and the temperature of evaporating alcohol from the resulted solutions. At a low amount of DSS (Zein-Que-DSS, 120:6:5), the proportion of β-sheet reasonably increased from 34.1 to 37.2%, together with the mild decrease of α-helix from 18.3 to 17.5%, and random coil from 27.6 to 25.1%. This could be due to the conversion of certain amounts of α-helices to β-sheets during aggregate formation mainly due to the unfolding of Zein. The proportion of α-helix and β-sheet gradually turned back to the original level of Zein nanoparticles (18.4% α-helix and 31.1% β-sheet) as the DSS increased. These findings suggest the restrictions in the conformational relaxation of Que-loaded Zein/DSS composites, which is in accordance with the fluorescence results.

**Figure 5:**
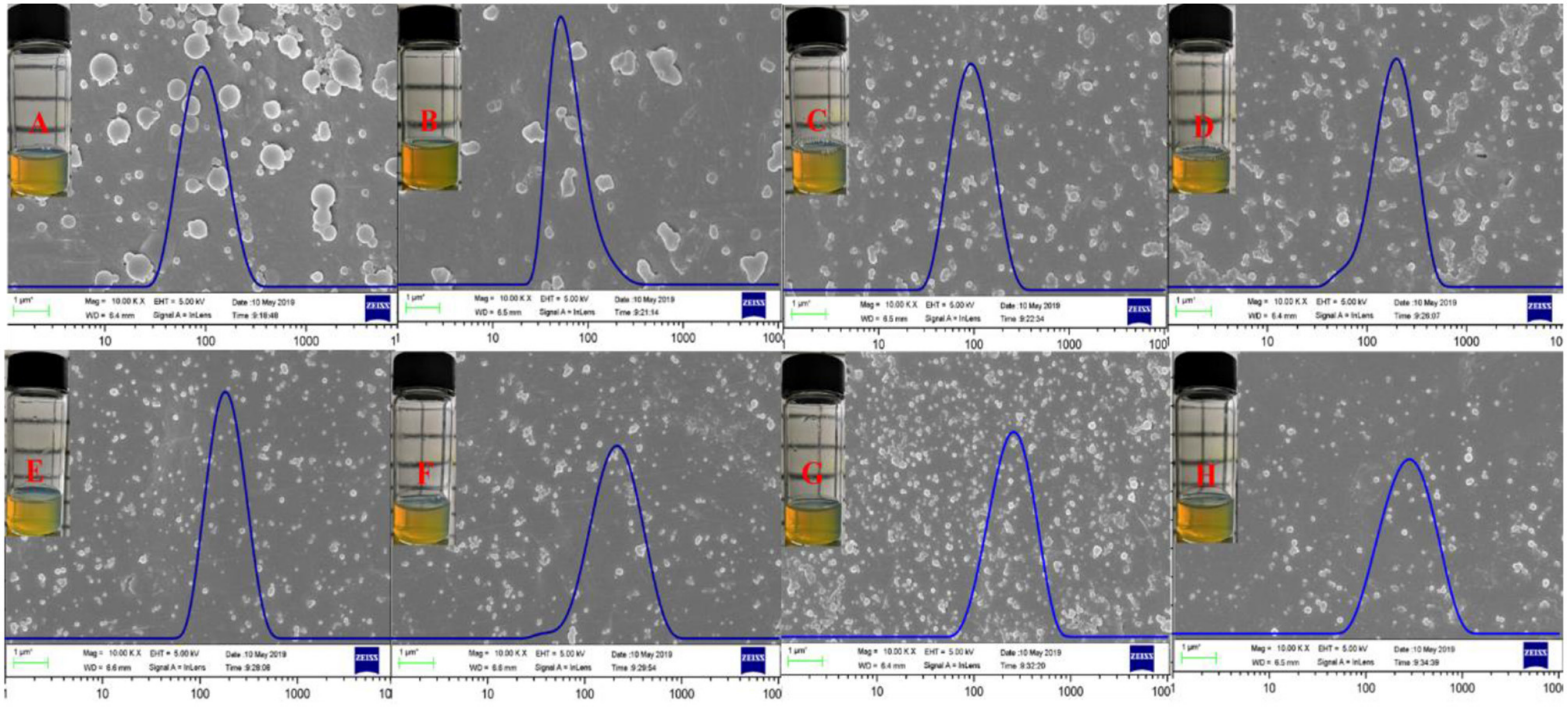
FE-SEM images, size distribution, and optical photography of Zein **(A)**, Zein-Que (120:6) **(B)** nanoparticles, and Zein-Que-DSS complex nanoparticles with different DSS concentrations [**(C)** Zein-Que-DSS (120:6:5); **(D)** Zein-Que-DSS (120:6:25); **(E)** Zein-Que-DSS (120:6:50); **(F)** Zein-Que-DSS (120:6:75); **(G)** Zein-Que-DSS (120:6:5); **(H)** Zein-Que-DSS (120:6:5)].

The FE-SEM images, granulometric distribution, and optical photography of the freshly prepared Zein nanoparticles and the Que-loaded Zein particles coated with different concentrations of DSS are shown in Figure 5. The average particle size calculated from the FE-SEM images (Figures 5A–H) through the direct measurements are as follows: 99.29, 84.68, 141.71, 112.86, 138.67, 134.36, 128.24, and 160.95 nm. These data are different from those results from DLS detection, which could be ascribed to the principle difference between the two methods. Zein nanoparticles are nanospheres with a particle size of approximately 90 nm and a smooth surface, which is in agreement with reported observations (Patel et al., 2012). Quercetin incorporation results in a relatively compact particle structure (Figure 5B) that tends to aggregate. Addition of DSS significantly improved the agglomeration. The optical photography of Que-loaded Zein complex nanoparticles with the presence or absence of DSS maintained a light yellow color in a narrow unimodal size distribution.

**Figure 6:**
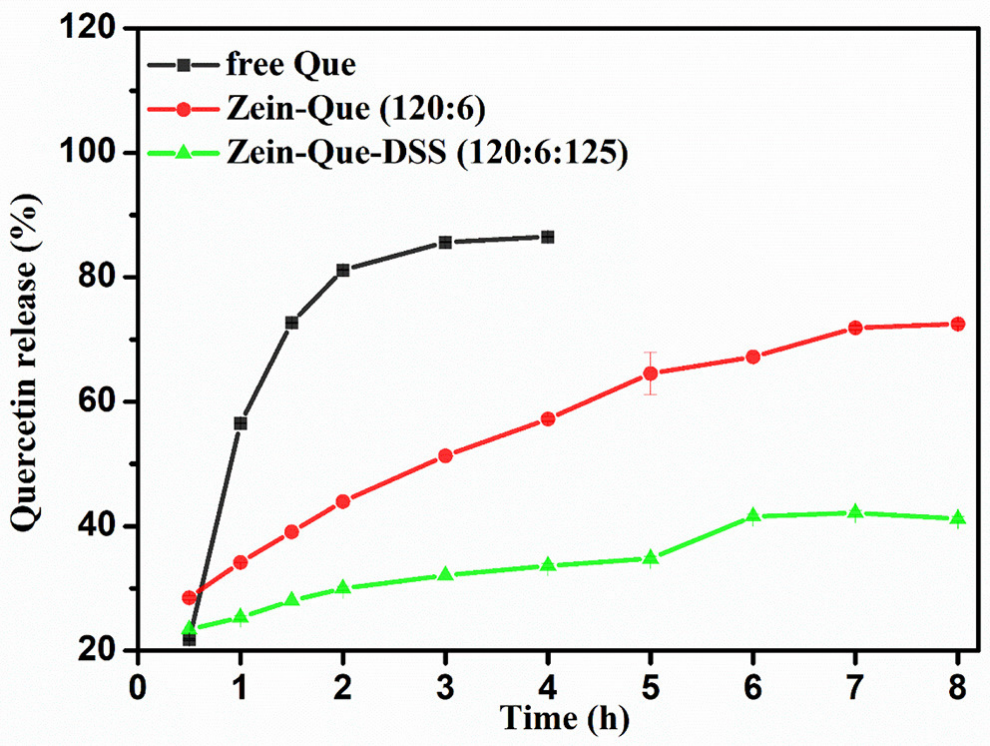
Kinetic release profile of free Que and Que obtained from Zein-Que (120:6) nanoparticles and Zein-Que-DSS (120:6:125) complex nanoparticles under stimulated intestinal conditions (pH = 6.4) at 37°C.

**Figure 7:**
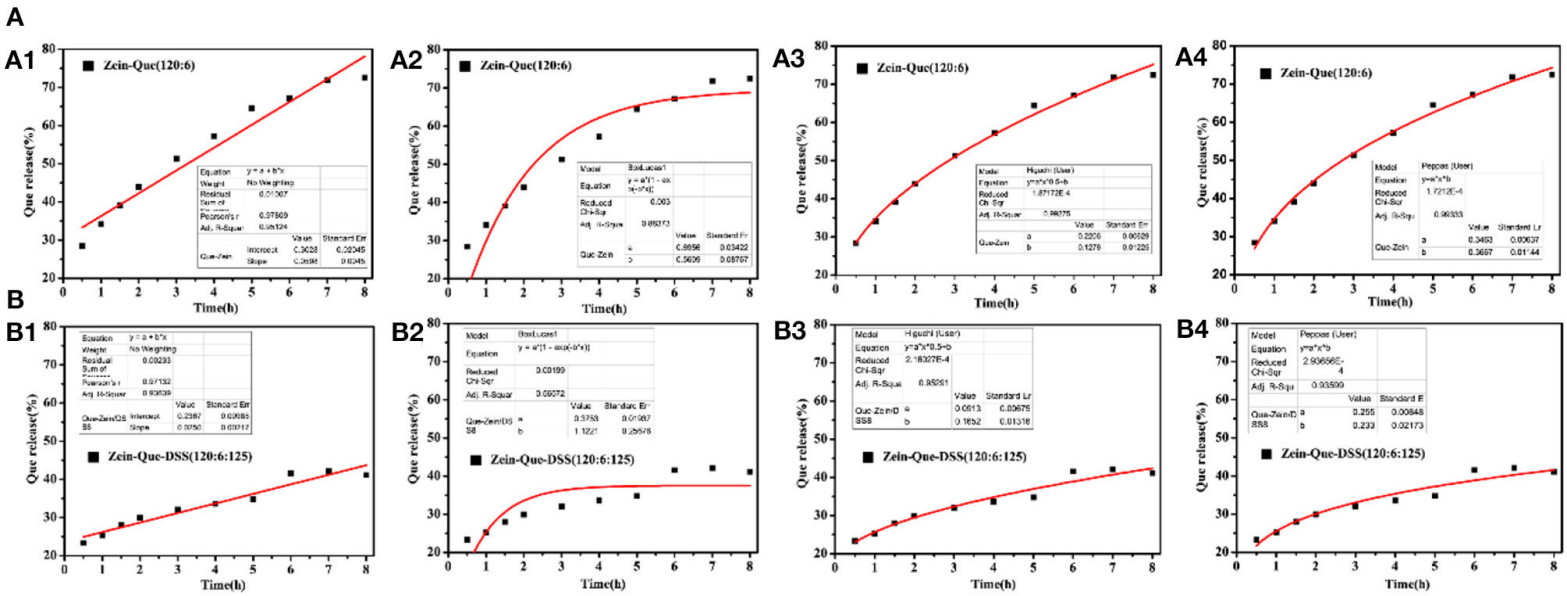
Que release fitting curve of the obtained nanoparticles and **(A)** Zein-Que (120:6) nanoparticles, **(B)** Zein-Que-DSS (120:6:125) complex nanoparticles; 1: Zero-order, 2: First-order, 3: Higuchi model, 4: Ritger-Peppas model.

Figure 6 illustrates the kinetic release profile of free-Que and Que obtained from Zein-Que (120:6) nanoparticles and Zein-Que-DSS (120:6:125) complex nanoparticles in a phosphate buffer solution (pH = 6.4). In the case of free-Que, a sharp release of Que has been observed during the initial 2 h with a total release rate of 81.2%. However, a more sustained release was noticed once Que was encapsulated in the Zein nanoparticles, which was further curtailed with the DSS coating on the nanoparticles. Consequently, the total Que release from Que-loaded Zein particles and Que-loaded Zein/DSS complex particles is found to be 43.95 and 29.94%, respectively. A similar trend was noticed even after 8 h release. Overall, DSS coating on the Zein nanoparticles has a positive effect toward obtaining sustained release of quercetin. Furthermore, Que release kinetics of Zein-Que (120:6) nanoparticles and Zein-Que-DSS (120:6:125) composite particles are reflected in Figure 7 using zero-order release kinetics, first-order release kinetics, the Higuchi model, and the Ritger-Peppas model, respectively. By comparing *R*^2^ value of several fitting curves, Que delivery of Que-loaded Zein nanoparticles (*R*^2^ = 0.951) and Que-loaded Zein/DSS composite particles (*R*^2^ = 0.936) were more in line with zero-order kinetics, namely, the controlled release pattern, wherein blood levels of Que would remain constant throughout the delivery period (Singhvi and Singh, 2011). Interestingly, Que release kinetics of Zein-Que (120:6) nanoparticles followed the Ritger-Peppas model (*R*^2^ = 0.993). In this model, the value of *n* indicates the release mechanism of Que. There are several simultaneous processes considered in the Ritger-Peppas model including diffusion of water into the microsphere and the dissolution of the polymer matrix. In the case of Que-loaded Zein nanoparticles, *n* < 0.45 (*n* = 0.37) corresponds to a Fickian diffusion mechanism. Fickian diffusional release occurs by the usual molecular diffusion of the drug due to a chemical potential gradient (Dash et al., 2010). High hydrophobicity of Zein is beneficial for delaying the release of Que. However, the hydrophilic residues at the protein surface (glutamine, glutamic acid, asparagine, and serine) are more sensitive to water, thus resulting in the extension of the Zein microsphere due to intestinal juice uptake. Furthermore, Que release of Zein-Que-DSS (120:6:125) composite particles is more in line with the Higuchi model (*R*^2^ = 0.953). This equation was used to describe the release of Que from hydrophilic matrix DSS in a time-dependent process based on the Fickian diffusion. Intestinal juice penetrates the sulfated polysaccharide and dissolves the Que, which then diffuses into the exterior environment. Once ingested, DSS coating of Que-loaded Zein/DSS composites swell and a gel layer forms on the microsphere surface. This gel layer retards further ingress of the intestinal fluid and subsequent Que release. It is well-revealed that Que release from ternary colloidal particles shows a typical time-dependent profile because of increased diffusion path length. The release of Que from composites involves the simultaneous penetration of the surrounding juice and leaching out of Que through interstitial channels or pores of the obtained nanoparticles.

**Figure 8:**
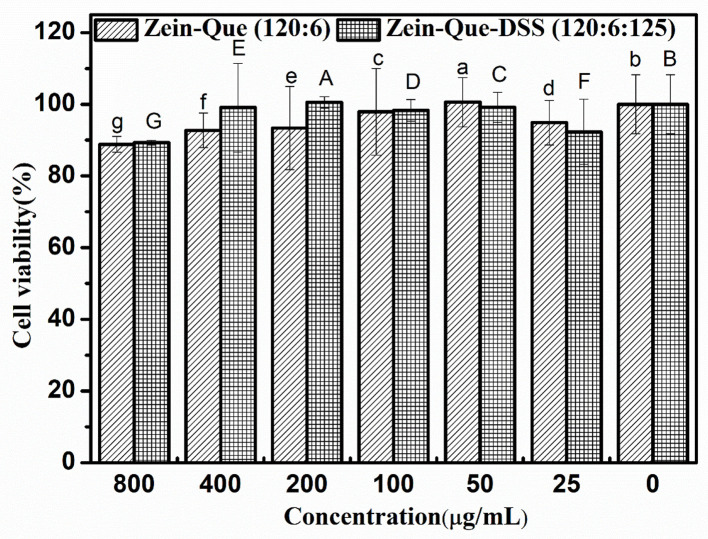
Cell viability of Caco-2 cells intervened by Zein-Que (120:6) nanoparticles and Zein-Que-DSS (120:6:125) complex nanoparticles with different concentrations. Different superscript letters in the same column represent significant differences (*p* < 0.05).

The Caco-2 cell lines were employed to establish the cytotoxicity of Que-loaded Zein microspheres and Que-loaded Zein/DSS complex nanoparticles. The proliferation ratio of all cultured cell lines incubated with different concentrations of Zein-Que particles and Zein-Que-DSS complex drugs for 24 h (Figure 8) is more than 88%, even at high levels of 800 μg/mL. Thus, Que-loaded Zein/DSS nanoparticles are safe with no cytotoxicity.

## Conclusions

The present work investigated the preparation, characterization, *in vitro* release, and cytotoxicity of quercetin-loaded Zein/dextran sulfate sodium biopolymeric nanoparticles. The self-assembly of ternary colloidal composites was accomplished through hydrophobic, hydrogen-bonding, and electrostatic interactions. Thus prepared nanoparticles have a size in the range of 180–250 nm, and display stability with high encapsulation efficiency. The nanoparticles are safe with no cytotoxicity for cellular uptake. The release mechanism of Que-loaded Zein/DSS (120:6:125) composites is in accordance with the Higuchi model (Q = 0.0913t^0.5^+0.1652, *R*^2^ = 0.953). The outcome not only promises favorable delivery of quercetin but could as well be applicable to widespread bioactive compounds.

## Data Availability Statement

All datasets generated for this study are included in the article/supplementary material.

## Author Contributions

TW conducted experiments and drafted the original manuscript. Research conception was initiated by XL along with gaining funding, writing, reviewing, and editing. LC and LL contributed to writing and reviewing. SJ participated in the research discussion, writing, reviewing, and editing. All authors contributed to the article and approved the submitted version.

## Conflict of Interest

The authors declare that the research was conducted in the absence of any commercial or financial relationships that could be construed as a potential conflict of interest.
